# Reading direction causes spatial biases in mental model construction in language understanding

**DOI:** 10.1038/srep18248

**Published:** 2015-12-15

**Authors:** Antonio Román, Andrea Flumini, Pilar Lizano, Marysol Escobar, Julio Santiago

**Affiliations:** 1 Mind, Brain, and Behavior Research Center, University of Granada, Spain

## Abstract

Correlational evidence suggests that the experience of reading and writing in a certain direction is able to induce spatial biases at both low-level perceptuo-motor skills and high-level conceptual representations. However, in order to support a causal relationship, experimental evidence is required. In this study, we asked whether the direction of the script is a sufficiente cause of spatial biases in the mental models that understanders build when listening to language. In order to establish causality, we manipulated the experience of reading a script with different directionalities. Spanish monolinguals read either normal (left-to-right), mirror reversed (right-to-left), rotated downward (up-down), or rotated upward (down-up) texts, and then drew the contents of auditory descriptions such as “the square is between the cross and the triangle”. The directionality of the drawings showed that a brief reading experience is enough to cause congruent and very specific spatial biases in mental model construction. However, there were also clear limits to this flexibility: there was a strong overall preference to arrange the models along the horizontal dimension. Spatial preferences when building mental models from language are the results of both short-term and long-term biases.

Can arbitrary and irrelevant aspects of the code that conveys a message, such as the directionality of its written script, modulate the mental representation of the contents of the message? In this paper we show that they do. It is now a standard assumption in the psychology of language comprehension that the final representation achieved by comprehenders is a mental model of the described situation^1^,^2^,^3^. Mental models are working memory representations about the world. They are analogical, spatial, and populated by concrete content, although they can also represent abstract content^4^,^5^. Once set up, mental models can be run in working memory, allowing us to anticipate consequences, reason, solve problems, and plan and perform actions. Language provides instructions that guide mental model construction in the comprehender^1^. For example, from a verbal description such as “the table is between the lamp and the TV”, the listener will construct a mental model that represents the spatial position of those three objects. However, the input leaves unspecified many aspects of the situation, which the comprehender must infer. In the present example, two different spatial arrangements of the three objects are consistent with the sentence: the lamp may be located to the left of the table or to its right (with the TV being situated at the opposite side). Jahn, Knauff, and Johnson-Laird^6^ observed that the preferred initial model for such a description aligns the three mentioned objects horizontally in left-to-right (L–R) order. They suggested that this preference for L–R models was a bias induced by the habitual reading and writing direction (RWD) of their German participants. As shown by Jahn *et al*.^6^, this bias is not inconsequential: the spatial arrangement of the objects in the mental model helps solving some kinds of problems and hinders others.

Román, El Fathi, and Santiago^7^ confirmed that such spatial biases correlate with habitual RWD as suggested by Jahn *et al*.^6^. They tested Spanish and Moroccan participants on a task that consisted in drawing the contents of auditorily presented sentences which described every-day elements arranged in static scenes (e.g., “The table is between the lamp and the TV”). Spanish participants showed a preference for drawing the lamp on the left and the TV on the right, whereas Moroccan participants (who read and write in Arabic, a language with a R-L script) tended to draw the lamp on the right and the TV on the left. These results add to a wide, though dispersed, literature, showing that habitual RWD correlates with lateral biases in a variety of mental processes and representations, including low level perceptual and attentional skills^8^,^9^,^10^,^11^,^12^,^13^, visual exploration^14^,^15^, motion preferences in drawing^16^,^17^,^18^, item choice from a list^19^, aesthetic preferences^20^,^21^,^22^, product attitudes in advertising^23^ and even kissing^24^. RWD also induces lateral biases in the mental representation of abstract concepts, such as number magnitude^25^,^26^, time^27^,^28^,^29^, events^30^,^31^, letter sequences^32^, and social groups differing in agentivity^33^. Studies conducted with illiterate participants^34^ show an absence of bias, consistently with the bias being caused by RWD.

How flexible are the lateral biases induced by RWD? Many studies in the literature have shown that preliterate children display either no lateral biases^30^, or L-R biases not linked to RWD^15^,^35^,^36^. As children learn to read, RWD-linked biases develop slowly and progressively^15^,^16^,^29^,^30^,^36^,^37^. Teaching children to read a second language with opposite directionality reduces those biases^15^,^38^, although they can be quite resistant to change when the new script is introduced at the adult age^39^. This pattern of results suggests that RWD induces spatial habits with a limited degree of flexibility, which needs important amounts of time and practice to develop and change.

In contrast, other studies have shown that these biases are very flexible, and that the mere exposition to a script can make its associated lateral biases to appear instantly in bilinguals^32^,^40^. Román *et al*.^7^ also tested a group of Moroccan bilinguals in either Standard Arabic or their second L-R language (either French or Spanish). The input language had a clear effect on mental model directionality, supporting a flexible deployment of spatial habits depending on the language in use. There was also a smaller influence of long-term habits linked to the participants’ higher practice in reading Arabic (favouring R-L biases). Thus, both short-term and long-term influences can be observed in the manifestation of lateral spatial biases, and it is still unclear what factors are responsible for the preponderance of one or another in a given situation (for a discussion see Román *et al*.^7^).

Unfortunately, most of the previous studies on RWD-related effects used correlational designs, comparing participants who read different scripts, which precludes random assignment of participants to groups. Therefore, extant studies do not allow us to establish a causal link between RWD and spatial biases, nor to isolate its time course during learning. The observed findings could be accounted for by a myriad of other factors that covary with script direction. For example, many cultural graphic manifestations, such as comic strips, calendars, and charts, covary in directionality with the script, and could account for the observed biases. The most solid conclusions can be drawn from studies that compare bidirectional bilinguals using each of their languages. However, this kind of participants constitute a special case, and the potential effects of modulating factors, such as the degree of bilingualism or the starting age, are still far from clear.

Training studies are better suited to reveal and explore causal effects. In such studies monolingual participants are randomly assigned to groups that practice scripts with different directionalities. This experimental design allows the establishment of causal relations between directional experience and spatial biases in target tasks, while all the other concurring factors are kept constant. To be clear, training studies can show that script directionality is a sufficient cause of changes in spatial biases. They cannot establish whether script directionality is a necessary cause, whether there are other causal factors in the spatial biases acquired by children during development in naturalistic situations, or how these other causes may interact with script directionality. However, establishing the status of script directionality as a sufficient cause of spatial biases is an important research goal. An additional advantage of training studies is that they also allow to measure the amount of training necessary for the development of the biases, as well as their course of decay.

To our knowledge, only two studies so far have followed this approach. Fischer, Mills, and Shaki^41^ showed that manipulating the associations of small and large numbers with the left and right sides of lines within a set of 20 cooking recipes (without changing script direction) was enough to change, and even revert, the SNARC effect. This study showed that some factors other than script direction may actually be able to induce spatial biases, and that their effects may develop quite fast. The only study which has directly manipulated script direction is Casasanto and Bottini^42^. Dutch participants presented with phrases like “one day later” were asked to judge whether the stimuli referred to the past or the future by pressing either a left or right key, or an up or down key. Text could be presented either in L-R direction, mirror reversed (R-L), or 90° rotated downwards (up-down, U-D) or upwards (down-up, D-U). After a short practice, mirror reading was able to reverse the standard association between left and past, and right and future, showing faster latencies for right-past and left-future responses than with the opposite mapping. When both text and response keys were rotated onto the vertical axis, U-D text induced an up/past-down/future congruency effect, and D-U text induced the reversed one. These seminal results support that script direction is a sufficient cause of biases in the directionality of the spatial representation of time, suggesting again that these directional habits can be established after a very short practice.

One central aim of the present study was to extend Casasanto and Bottini’s^42^ results to a task that taps onto the processes of mental models construction from linguistic inputs. This task is a variant of the drawing task used by Román *et al*.^7^. First of all, participants had to read a short Spanish text in either standard (L-R), mirror reversed (R-L), or 90° rotated print, either upwards (D-U) or downwards (U-D). Then, descriptions of static scenes involving geometrical shapes, such as “the square is between the cross and the triangle”, were presented auditorily. Finally, participants were asked to draw the three objects on a sheet of paper, in order to assess whether and how the directionality of their drawings was influenced by the prior reading experience. The drawing task used geometrical shapes instead of real world objects as in Román *et al*.^7^, so that participants would not feel necessarily compelled to arrange them along the horizontal axis.

## Methods

### Participants

One hundred Spanish psychology students at the University of Granada (mean age 21.9 years; 14 males; 5 left-handed). All of them were native Spanish speakers and did not know any language with a different RWD. Informed consent was obtained from all participants. The procedure of the study was approved by the Ethics Committee of the University of Granada and it was carried out in accordance with the approved guidelines.

### Materials

For the reading task, we prepared a 1195 words fiction narrative in Spanish. Words were printed in 15 points Arial font. The text occupied four pages; each one contained five to six paragraphs of four to six lines each one. For the drawing task we selected nine common geometrical shapes which could be drawn easily (square, rectangle, cross, rhombus, triangle, circle, trapezium, oval, pentagon). As a result of combining the names of those geometrical shapes, 441 sentences describing a between relation among three different shapes were constructed. For example, “The circle is between the cross and the rectangle” or “The oval is between the triangle and the rhombus”. All sentences referred to completely static scenes without any agentive structure. From this set of 441 sentences, 40 sentences were randomly selected to be used in the task (see Appendix). They were randomly divided into two lists of 20 sentences each one. Each participant was presented with only one list. The sentences were read aloud by a female experimenter and recorded in independent sound files.

### Procedure

**Figure 1:**
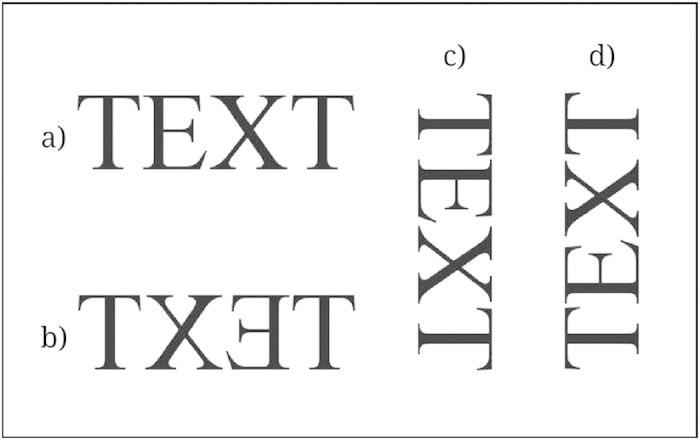
(**a**) Standard L-R text; (**b**) Mirror R-L text; (**c**) Downwards U-D text; (**d**) Upwards D-U text.

The participant sat in front of a computer screen at a desk with a pen and a stack of 20 blank square sheets. Stimulus presentation was controlled by Eprime 2.0. Participants were instructed that they should read aloud a 4-page text presented on the screen at their own pace. Reading aloud secured that the text was read. Instructions warned them to pay attention to the text because at the end of the experiment there would be five questions about the content of the story. Each group of 20 participants read the text with a different directionality (see Fig. 1): L-R (standard), R-L (mirror reversed), U-D (rotated 90° clockwise), and D-U (rotated 90° counterclockwise). There was also a control group with 20 participants that performed the drawing task without the prior reading phase. After finishing reading, they moved on to draw a set of auditorily presented sentences, each one on a different sheet. This task was presented as a filler task before the final comprehension questions. Care was exercised not to mention any particular spatial arrangement (e.g., horizontal) for the drawings. The program presented the sentences through loudspeakers. Participants controlled the rate of presentation by pressing a button to advance to the next one.

### Data coding

During the drawing task, the experimenter stood behind the participant and coded *in situ* and out of the participant’s sight the order in which each of the three mentioned objects was drawn (object order) and the order in which participants completed the different spaces in the sheet: right, left, center, up, down or any other (spatial order). Finally, model order was coded a posteriori from the drawings themselves, depending on the locations where the two side objects were placed with respect to the central object (for more detail, see the Data Coding section in Roman *et al*.^7^). As discussed in Román *et al*.^7^, the three measures (object order, spatial order, and model order) are linked, such that the use of a given object and spatial order determines a final model order. For example, when the three objects mentioned in the sentence “the square is between the triangle and the cross” are drawn in order 123 (the same order of mention, starting with the square, followed by the triangle, and then the cross) and in the spatial order CLR (filling up first the central position, then the left and then the right position), the resulting model is necessarily a L-R model (“triangle - square - cross”). Román *et al*.^7^ observed a strong tendency to draw objects in the same order as they are mentioned in the sentence (58% of trials in the Spanish readers group) with all the other orders being preferred to the same (low) extent (betwen 2.9 and 16.6%). Spanish (L-R) and Moroccan (R-L) readers showed the same pattern, suggesting that this preference is caused by a shared strategy devoted to minimize working memory load. Therefore, the differences in preferred mental model directionality arose from differences in the order in which the spatial slots were filled, with the order CLR being modal in the Spanish group (42.5%), and the order CRL being modal in the Moroccan group (43.4%). Román *et al*.^7^ thus showed that analyzing spatial order and object order independently did not qualify the results obtained in the measure of model order, so in the present study we focused our anlysis only on the latter. However, as a control check, we also report the analysis of object order in all five groups.

### Design and Data Analysis

There were four experimental groups of 20 participants, depending on the directionality of the training: L-R (standard), R-L (mirror reversed), U-D (rotated 90° clockwise), and D-U (rotated 90° counterclockwise). Furthermore, we run a control group of 20 participants with no reading phase to serve as a baseline. In each condition, the number of drawings with a L-R, R-L, U-D and D-U directionality was counted. Because the four conditions are not independent, it was not possible to use ANOVA. Therefore, we computed binomial logistic regressions. Due to the categorical and multinomial nature of our dependent variable, it would be appropriate to apply a mixed-effect multinomial regression, since participants can produce only four qualitatively different responses (horizontal left, horizontal right, vertical up or vertical down). However, to simplify the analysis and to have the freedom to focus on the contrasts in which we are interested, we carried out binomial logistic regressions in which the relevant response alternatives were pooled into only two contrasting kinds. This requires dummy coding our dependent variable (percentage of responses in each direction) into a dichotomous variable where, for each of the 4 possible responses, the direction chosen by the subject is set to “1” and the rest are assigned the value “0”. Thus, we could test, for example, whether the proportion of RL responses (dummy coded as 1) after reading mirror-reversed text is higher than after reading standard text. If these ratios differ it will be reflected in the odds ratio taking values away from “1”. For each relevant contrast we report Wald,s χ^2^ statistic, the odds ratio and its confidence intervals. We also report effect sizes using both Hedges,g and the Common Language (CL) effect size, which indicates the probability that, for any randomly selected pair of individuals, the score of the person from one group is higher than the score of the person from the other group^43^. We were also interested in analyzing the time course of the decay of the effect of prior reading experience. Thus, we analyzed the percentage of LR models for each of the 20 trials of the drawing phase. We expected the effect to be largest at the beginning of the drawing phase and to fade progressively away.

### Open materials and data

** All materials, programs and raw data can be obtained from Open Science Foundation at http://osf.io/v2n5x.

## Results

If the central object (e.g., the circle in the sentence “The circle is between the cross and the rectangle”) was drawn anywhere else than at the center, the trial was considered invalid and was not included in the final analysis. Trials where it was not possible to ascertain the axis along which the objects were drawn (i.e., diagonal arrangements) were also discarded. The number of items rejected was less than 2%. Left-handers were few (five) and unequally distributed between groups, so the effect of handedness could not be assessed. The results did not change in any relevant way if left-handers were removed from the data. The average time (in seconds) of the previous reading phase for each condition was: Control = 0 s; L-R = 421 s; R-L = 1820 s; D-U = 433 s; U-D = 512 s. The average time of the final drawing phase for each condition was: Control = 321 s; L-R = 313 s; R-L = 282 s; D-U = 305 s; U-D = 265 s.

**Figure 2:**
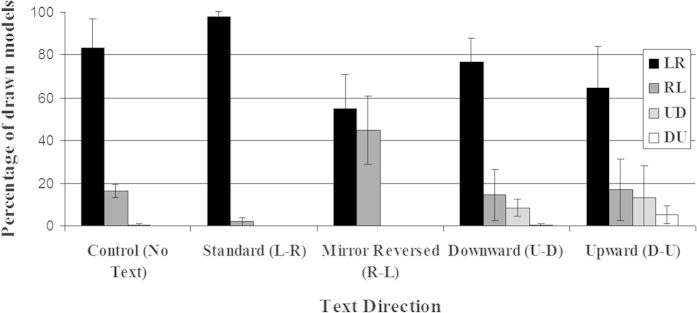
Proportion of drawn models of each directionality (L-R, R-L, U-D, and D-U) in each training group. Error bars show 95% confidence intervals.

The control group (drawing task without prior reading of the text) showed 83.5% of L-R models. This percentage is similar to that found in the Spanish participants of Román *et al*.^7^, which amounted to 70.7%, and shows the default effect of native RWD. The results revealed clear effects of the prior reading phase (see Fig. 2). All the conclusions that can be drawn from visual inspection of confidence intervals in Fig. 2 were supported by binomial logistic regressions.

In the group who read standard L-R Spanish, there were no vertically oriented drawings. Most drawings (98.2%) were arranged along the horizontal axis, flowing from left to right. The remaining drawings (1.8%) were horizontal from right to left, and did not differed significantly from zero (see Fig. 2; *t*(19) = −1.95, *p* = 0.07). The L-R group scored significantly higher in L-R models (*M* = 98.2%, *SD* = 4.15) than the control group (*M* = 83.5%, *SD* = 27.63; Hedges’s *g*_*s*_ = 0.73, *CL* = 0.70; Wald *χ*^*2*^ = 31.4, *p* < 0.001, *Odds ratio* = 9.79, 95% *CI* = [4.41, 21.74]).

By contrast, in the group who read mirror reversed R-L text, the percentage of L-R drawings decreased to 55.1% while R-L drawings increased to 44.9%. Again, there were no drawings with vertical directionality. The number of L-R models was lower in the R-L group (*M* = 55.1%, *SD* = 32.28) than in the control group (*M* = 83.5%, *SD* = 27.63; Hedges’s *g*_*s*_ = 0.93, *CL* = 0.75; Wald *χ*^*2*^ = 76.4, *p* < 0.001, *Odds ratio* = 4.6, 95% *CI* = [3.27, 6.48]). This number was also clearly smaller than in the L-R group (*M* = 98.2%, *SD* = 4.15; Hedges’s *g*_*s*_ = 1.84, *CL* = 0.91; Wald *χ*^*2*^ = 93.15, *p* < 0.001, *Odds ratio* = 45.04, 95% *CI* = [20.79, 97.60]). Therefore, a brief experience of reading in the usual L-R or opposite R-L direction was able to affect the directionality of mental models built from auditorily presented sentences.

The two groups with previous exposure to horizontal reading (L-R and R-L) showed a very strong preference to locate the drawn models on the horizontal axis, as not a single vertical drawing was produced. Would previous experience with a vertical script change this preference? As shown in Fig. 2, the U-D group also showed a predominance of L-R models (*M* = 76.9%, *SD* = 22.4), which was significantly lower than the Control group (Wald *χ*^*2*^ = 7.9, *p* = 0.005, *Odds ratio* = 1.68, 95% *CI* = [1.17, 2.42]). The number of L-R models in the U-D group was also below the level of the L-R group (*M* = 98.2%, *SD* = 4.15; Hedges’s *g*_*s*_ = 1.29, *CL* = 0.82; Wald *χ*^*2*^ = 49.2, *p* < 0.001, Odds ratio = 16.49, 95% *CI* = [7.54, 36.09]), but above the level of the R-L group (*M* = 55.1%, *SD* = 32.28; Hedges’s *g*_*s*_ = 0.77, *CL* = 0.71; Wald *χ*^*2*^ = 41.52, *p* < 0.001, *Odds ratio* = 2.73, 95% *CI* = [2.01, 3.7]). The percentage of R-L models in the U-D group (*M* = 14.5%, *SD* = 23.9) was also at the same level as the control group (*M* = 16.2%, *SD* = 27.8; Wald *χ*^*2*^ = 0.015, *p* = 0.90, *Odds ratio* = 1.025, 95% *CI* = [0.69, 1.52]), below the level of the R-L group (*M* = 44.9%, *SD* = 32.28; Hedges’s *g*_*s*_ = 1.05, *CL* = 0.77; Wald *χ*^*2*^ = 81.3, *p* < 0.001, Odds ratio = 4.81, 95% *CI* = [3.42, 6.77]) and above the level of the L-R group (*M* = 1.8%, *SD* = 4.15; Hedges’s *g*_*s*_ = 0.73, *CL* = 0.7; Wald *χ*^*2*^ = 30.2, *p* < 0.001, Odds ratio = 9.36, *CI* = [4.21, 20.78]). Overall, the proportion of L-R and R-L models in the U-D group moves closer to the values of the control group. In the case of L-R models, understandably it is slightly below the control group because the fixed total number of responses must be shared among a greater number of alternatives. Thus, some practice with U-D reading did not change dramatically the proportion of L-R and R-L models as compared to a situation of no prior reading.

However, previous experience reading U-D text was indeed able to increase U-D models from 0% to 8.3%, a numerically small but significant change, as assessed by a t-test against zero (see Fig. 2; *t*(19) = 4.51, *p* = 0.0002). In contrast, the percentage of D-U models was not significantly different from zero in this group (*t*(19) = 1, *p* = 0.33). In short, reading U-D script selectively increases congruent U-D models.

In the group with prior experience reading D-U text, the number of L-R models (*M* = 64.53%, *SD* = 39.19) fell below the L-R group (*M* = 98.2%, *SD* = 4.15; Hedges’s *g*_*s*_ = 1.18, *CL* = 0.80; Wald *χ*^*2*^ = 72.2, *p* < 0.001, Odds ratio = 28.9, 95% *CI* = [13.3, 62.9]), and even below the control group (Wald *χ*^*2*^ = 37.1, *p* < 0.001, Odds ratio = 2.95, 95% *CI* = [2.08, 4.19]), and the U-D group (Wald *χ*^*2*^ = 12.4, *p* < 0.001, Odds ratio = 1.75, 95% *CI* =[1.28, 2.40]). Again, the decline in L-R responses can be due to increased numbers in other directions. In fact, the D-U group is the one with more kinds of different responses. The amount of R-L models (*M* = 17.1%, *SD* = 29.0) was similar to that in the control group (*M* = 16.2%, *SD* = 27.8; Wald *χ*^*2*^ = 0.22, *p* = 0.64; Odds ratio = 1.1, 95% *CI* = [0.74, 1.62]) and U-D group (*M* = 14.5%, *SD* = 23.9; Wald *χ*^*2*^ = 0.36, *p* = 0.55, Odds ratio = 1.13, 95% *CI* = [0.76, 1.66]). In contrast, it was greater than in the L-R group (*M* = 1.8%, *SD* = 4.14, Hedges’s *g*_*s*_ = 0.72, *CL* = 0.70; Wald *χ*^*2*^ = 33.7, *p* < 0.001, Odds ratio = 10.54, 95% *CI* = [4.76, 23.32]) and smaller than in the R-L group (*M* = 44.9, *SD* = 32.28; Hedges’s *g*_*s*_ = 0.89, *CL* = 0.74; Wald *χ*^*2*^ = 72.5, *p* < 0.001, Odds ratio = 4.27, 95% *CI* = [3.06, 5.97]).

Reading D-U text did increase D-U models significantly above zero (*t*(19) = 2.77, *p* = 0.01). Moreover, there was also a trend toward increasing the proportion of U-D models above zero (*t*(19) = 1.97, *p* = 0.06). Therefore, the data suggest that the effect of reading D-U text was somewhat less specific than the effect of reading U-D text, leading to a clear, though small, increment of D-U models but also to a slightly elevated proportion of U-D models.

**Figure 3:**
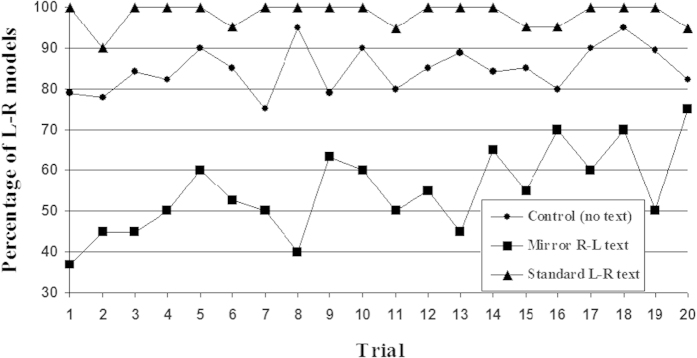
Percentage of L-R models over trials in the L-R, R-L and control groups.

The analysis of the effect throughout the drawing trials reveals a progressive decay. Figure 3 shows the percentage of L-R models in the L-R, R-L and control groups. A t-test of the slope of the regression line for each condition shows a slope different from zero in the R-L group (*t*(19) = 3.73, *p* = 0.001), but neither in the control group (*t*(19) = 1.65, *p* = 0.12) nor in the L-R group (*t*(19) = 0.06, *p* = 0.95).

As a final control check, we report the percentages of choice of each object order in each group in Table 1. All groups showed equally strong preference for object order 123 (i.e., drawing objects in the same order as they are mentioned in the sentence), thereby replicating prior findings by Román *et al*.^7^. Actually, the preference for object order 123 in the current study (*M* = 81.9%) seems to be greater than in the Spanish group of Román *et al*.^7^ (*M* = 58%). The reasons for this difference are at present unclear, but it may be related to the use of objects with more difficult names in the present study (geographical shapes versus common objects). The preference for object order 123 is likely due to a strategy that seeks to minimize working memory load. The use of this strategy in all groups and to the same extent therefore suggests that the observed differences in mental model directionality across the groups are due to the order in which the positions on the paper are filled, as also pointed out by Román *et al*.^7^.

## Discussion

In the present study, a group of participants listened to spoken sentences of the kind “The triangle is between the square and the circle” and drew the described scenes. Four other groups carried out the same task after reading a short text with either L-R (standard), R-L (mirror reversed), U-D (downwards) or D-U (upwards) script. The directionality of their drawings revealed both long-term spatial biases, stable and difficult to change, as well as short-term, highly flexible biases. Firstly, there was a strong preference to construct mental models along the horizontal versus the vertical axis. This was observed in all groups, including those that read vertically oriented text, even though the geometrical figures could be drawn just as well along the vertical axis. This preference for horizontal models may have different and non-exclusive sources. It may be the result of long-term experience with a horizontal script, of universal preferences of mental model construction, or biomechanical factors like locomotion in the horizontal plane. In fact, people may have the tendency to place the objects in a mentally simulated scene as if they were resting on the ground instead of piled up in a stack, which is probably a less frequent arrangement in the everyday experience of the world. Future studies using readers of vertical scripts, such as Taiwanese Chinese, can help disentangle these possible causes.

Horizontal models also revealed a clear preference for arranging the elements from left to right, as it was also observed in the Spanish group of Roman *et al*.^7^. This supports previous evidence about a strong default L-R bias for Spanish readers, which may also result from two sources: the long-term experience with a L-R script, and the universal tendencies that favour L-R processing. This latter possibility is supported also by several prior studies showing that the L-R bias in L-R readers is stronger than the R-L bias in R-L readers (as reported by Román *et al*.^7^ in a task analogous to the present one; see the Introduction for references using other methodologies).

Can long-term biases be affected by a short reading experience? The present study provides a positive answer to this question. A short practice reading in each of the four directions was able to induce quite specific spatial biases on drawings, which followed the same direction of the prime text. Reading mirror reversed R-L text increased considerably the amount of R-L models, while reading U-D and D-U texts was also able to significantly increase (above zero) the amount of U-D and D-U models, respectively, although the size of this increase was numerically small. Given the absolute absence of vertical models in the control group, as well as in the two horizontal reading groups, this small increase is no doubt of theoretical relevance. Finally, even when the prime text was presented in the standard L-R fashion, it was able to intensify the default L-R bias, suggesting that even pre-established, life-long L-R tendencies can be strengthened by immediate practice.

The present results suggest that the amount of practice needed to substantially change previously established tendencies is very small: less than 10 minutes (except in the mirror R-L condition, which took about 30 minutes) of reading a text with a different directionality sufficed to affect spatial preferences in mental models construction. This is in agreement with the study by Casasanto & Bottini^42^, but contrasts sharply with many studies that document a slow and progressive development of spatial biases during the process of learning to read (e.g., see^15^; see review in the Introduction section) and suggests the implication of different mechanisms underlying short-term and long-term biases. The present findings also showed that short-term biases are short-lived, and may vanish after a period of a few minutes. It is possible that repeated, consistent experiences will produce short-term biases that progressively return to lower asymptotes, leading to the modification of prior long-term biases, or to the establishment of different ones.

In our view, the two types of biases (short and long-term) are two sides of the same coin. Both have a great adaptive value and are necessary for a good performance in an environment which is both stable and constantly changing. We need, on one hand, an extremely flexible system that can adapt to sudden changes in our experience. On the other hand, we need a long-term memory system where adaptations to persistent changes can progressively crystalize, avoiding the need of relearning the new conditions. Future studies using training designs providing more extended practice should be able to trace the time course of the establishment of long-term biases in detail.

Present results, as well as those in Román *et al*.^7^, are consistent with the following process model of the implementation of spatial biases induced by reading direction. When understanding sentences like “the A is between the B and the C”, participants overwhelmingly use a strategy aimed at minimizing working memory load, such that objects are drawn in the same order as they were mentioned in the sentence. Thus, in most trials their performance can be explained by the use of a phonological buffer in which they maintain the just heard sentence in verbatim form by a process of repetitive rehearsal (akin to the phonological loop proposed by Baddeley and Hitch^44^). As they rehearse the sentence, the mentioned objects are included in the mental model and drawn on paper in the same order as they appear in the sentence. The first-mentioned reference object is drawn first at the central location. It is when the second object is rehearsed and placed in the model and on the paper when a decision must be made about its location with respect to the central object. At this moment, prior reading experiences bias participants’ choices, both regarding the spatial axis (horizontal or vertical) and the side on that axis. The location of the third object is then chosen by placing it at the opposite end of the axis on which the second object stands.

To conclude, the present findings provide clear support for a causal influence of RWD on the spatial inferences that are made during mental model construction from auditory linguistic input. It remains to be determined whether RWD is the one and only source of directional biases, as the same directional training can also be obtained from experiences with comic strips, book pages, number lines, charts, etc. The present study clearly establishes that exposure to text is a sufficient cause, in and by itself, of change in spatial biases, but does not discard other potential concurring causes.

## Additional Information

**How to cite this article**: Román, A. *et al*. Reading direction causes spatial biases in mental model construction in language understanding. *Sci. Rep*. **5**, 18248; doi: 10.1038/srep18248 (2015).

## Supplementary Material

Supplementary Information

## Figures and Tables

**Table 1:** Percentage of choice of each object order in each group.

Object order	Control	L-R	R-L	U-D	D-U
123	82.0	79.0	82.9	85.2	80.3
132	0	5.4	8.3	6.3	7.7
213	10.7	6.9	1	1.8	6.9
231	7.3	7.9	7.1	5.8	3.6
312	0	0	0	0.5	0.3
321	0	0.8	0.8	0.5	1.3

Order 123 means that the object mentioned first is drawn first, the object mentioned second is drawn second, and the object third is drawn in third place. Order 312 means that the object mentioned third in the sentence is drawn first, followed by the object mentioned in first place and, finally, the object mentioned in second place.
