# Supplementary material for: Reading direction causes spatial biases in mental model construction in language understanding
Source: Sci Rep. 2015 Dec 15;5:18248. doi: 10.1038/srep18248 (PMC4678875; doi:10.1038/srep18248)
Supplement: Supplementary Information [file srep18248-s1.pdf]

Appendix of Román, Flumini, Lizano, Escobar & Santiago, "Reading direction causes spatial biases in mental model construction in language understanding".

Sentences used in the drawing task.

Nº	List A	List B
1	The circle is between the cross and the rectangle	The square is between the rhombus and the cross
2	The oval is between the triangle and the rhombus	The triangle is between the pentagon and the oval
3	The pentagon is between the trapezium and the square	The oval is between the trapezium and rhombus
4	The trapezium is between the circle and the square	The rectangle is between the square and the circle
5	The pentagon is between the triangle and the oval	The pentagon is between the rhombus and the trapezium
6	The cross is between the rhombus and the rectangle	The oval is between the pentagon and the circle
7	The rhombus is between the pentagon and the trapezium	The pentagon is between the oval and the rectangle
8	The cross is between the rectangle and the oval	The cross is between the rectangle and the square
9	The triangle is between the circle and the square	The circle is between the trapezium and triangle
10	The trapezium is between the rhombus and the oval	The trapezium is between the square and the pentagon
11	The pentagon is between the triangle and the cross	The cross is between the circle and the rectangle
12	The circle is between the rectangle and the square	The trapezium is between the oval and the square
13	The pentagon is between the oval and the circle	The square is between the trapezium and circle
14	The trapezium is between the triangle and the rhombus	The rhombus is between the rectangle and the cross
15	The rectangle is between the square and the cross	The triangle is between the rhombus and oval
16	The oval is between the rectangle and the	The triangle is between the pentagon and the

	pentagon	cross
17	The triangle is between the circle and the trapezium	The rectangle is between the cross and the oval
18	The cross is between the square and the rhombus	The rhombus is between the rectangle and the pentagon
19	The square is between the trapezium and the oval	The trapezium is between the rhombus and triangle
20	The pentagon is between the rhombus and the rectangle	The square is between the triangle and the circle
